# Validation of *PDE9A* Gene Identified in GWAS Showing Strong Association with Milk Production Traits in Chinese Holstein

**DOI:** 10.3390/ijms161125976

**Published:** 2015-11-05

**Authors:** Shao-Hua Yang, Xiao-Jun Bi, Yan Xie, Cong Li, Sheng-Li Zhang, Qin Zhang, Dong-Xiao Sun

**Affiliations:** College of Animal Science and Technology, Key Laboratory of Animal Genetics and Breeding of Ministry of Agriculture, National Engineering Laboratory of Animal Breeding, China Agricultural University, Beijing 100193, China; yangshaohua309@126.com (S.-H.Y.); yaning812@126.com (X.-J.B.); xiey.300822@aliyun.com (Y.X.); li10020902@163.com (C.L.); zhang62733697@163.com (S.-L.Z.); qzhang@cau.edu.cn (Q.Z.)

**Keywords:** dairy cattle, *PDE9A*, milk production traits, association analysis, GWAS, gene expression

## Abstract

Phosphodiesterase9A (*PDE9A*) is a cyclic guanosine monophosphate (cGMP)-specific enzyme widely expressed among the tissues, which is important in activating cGMP-dependent signaling pathways. In our previous genome-wide association study, a single nucleotide polymorphism (SNP) (BTA-55340-no-rs^b^) located in the intron 14 of *PDE9A*, was found to be significantly associated with protein yield. In addition, we found that *PDE9A* was highly expressed in mammary gland by analyzing its mRNA expression in different tissues. The objectives of this study were to identify genetic polymorphisms of *PDE9A* and to determine the effects of these variants on milk production traits in dairy cattle. DNA sequencing identified 11 single nucleotide polymorphisms (SNPs) and six SNPs in 5′ regulatory region were genotyped to test for the subsequent association analyses. After Bonferroni correction for multiple testing, all these identified SNPs were statistically significant for one or more milk production traits (*p* < 0.0001~0.0077). Interestingly, haplotype-based association analysis revealed similar effects on milk production traits (*p* < 0.01). In follow-up RNA expression analyses, two SNPs (c.-1376 G>A, c.-724 A>G) were involved in the regulation of gene expression. Consequently, our findings provide confirmatory evidences for associations of *PDE9A* variants with milk production traits and these identified SNPs may serve as genetic markers to accelerate Chinese Holstein breeding program.

## 1. Introduction

Milk production traits are the most economically important traits controlled by numerous genes and environmental factors in dairy cattle. Therefore, an improvement in milk production traits continues to be the most profitable breeding objective. Mutations can alter breeding values of economical traits in dairy cattle [1]. New molecular techniques focused on genome analysis have made it feasible to screen for mutations associated with complex traits by genome-wide association study (GWAS) [2]. Compared with traditional QTL (quantitative trait loci) mapping strategy, GWAS shows obvious advantages both in the power to detect harboring variants and in simplifying the discovery of causal variants [3]. Thus, GWAS have been widely recognized as an important strategy to explore genes associated with complex traits in many species. Using Illumina 50K chip, we identified 105 genome-wide significant SNPs associated with milk production traits in Chinese Holstein population. Among these SNPs, a SNP, BTA-55340-no-rs^b^ (*p* = 9.72 × 10^−7^, *n* = 1815), located in the intron 14 of the *phosphodiesterases 9A* (*PDE9A*) gene was highly significant with effects on protein yield [4]. The *PDE9A* gene is located on bovine BTA 1, which included a number of identified QTLs for milk production traits [5,6]. These findings strongly suggest that *PDE9A* may be one of the most promising novel candidates for milk production traits. 

*PDEs*, a large family of enzymes specifically hydrolyzing the second messenger cAMP and/or cGMP, are the only cellular mechanism for degrading cAMP and cGMP [7] and thus play a critical role in regulating the intracellular levels of these second messengers and subsequently functional responses of cells [8,9,10,11]. The *PDE9A* gene encodes a cGMP-specific high-affinity *PDE* that seems to be widely expressed among tissues or organs in humans and mice [12,13]. The bovine *PDE9A* gene has 19 exons spanning 110 kb. According to the significant evidences in our GWAS as well as comparative genomics, we therefore assumed that the *PDE9A* gene could be a positional and functional candidate gene for milk production traits in Chinese Holstein population.

As a novel candidate for milk composition traits detected by GWAS, however, the bovine *PDE9A* gene has not been reported in relation with milk production traits. Therefore, the purpose of the present study was to search for potential casual genetic variants associated with milk production traits in an independent dairy cattle population. Furthermore, tissue expression pattern and the role of regulatory SNPs on their mRNA expression were also investigated. The results showed that the two identified variants in 5′ regulatory region of *PDE9A* may be important genetic factors involved in milk production ability and can be used in marker-assisted breeding on the basis of further validation.

## 2. Results and Discussion

### 2.1. Molecular Evolutionary Analysis of the Bovine PDE9A Gene

Based on protein sequence of bovine *PDE9A* (XP_005202092.1), we obtained the following similarities by comparing of the *PDE9A* amino acid sequence with seven other animal species (Table 1): *Homo sapiens* (85.39%), *Rattus norvegicus* (90.52%), *Mus musculus* (91.33%), *Gallus gallus* (81.64%), *Danio rerio* (77.81%), *Capra hircus* (98.81%) and *Ovis aries* (98.11%). The results suggested that the *PDE9A* gene was extremely conserved within mammalian species.

**Table 1:** Comparative analysis of *PDE9A* amino acid sequence of different species.

Species	GenBank Accession	Similarity
*Capra hircus*	XP_005675698.1	98.81%
*Ovis aries*	XP_004003956.1	98.11%
*Mus musculus*	NP_001157220.1	91.33%
*Rattus norvegicus*	XP_008771017.1	90.52%
*Homo sapiens*	NP_001001567.1	85.39%
*Gallus gallus*	XP_003640550.1	81.64%
*Danio rerio*	XP_009302929.1	77.81%

**Figure 1:**
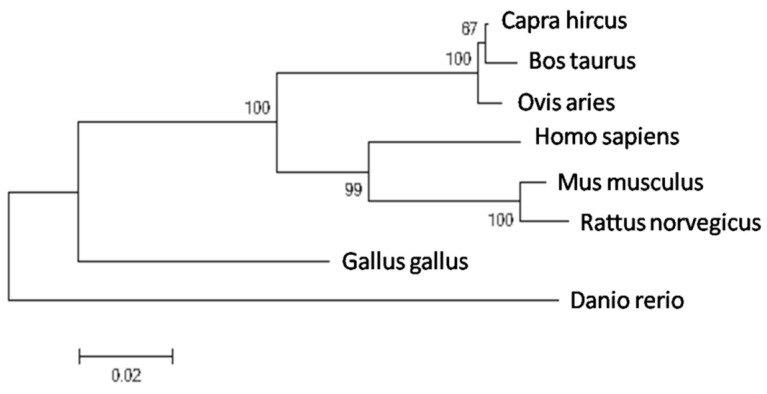
Phylogenetic tree of the *PDE9A* gene in different species. Numbers at each branch showed the percentage based on neighbor joining.

A phylogenetic tree was constructed using the MEGA 4.0.2 software to better understand the potential evolutional process. According to the phylogenetic tree (Figure 1), the bovine *PDE9A* was found to be phylogenetically closest to *Capra hircus* and *Ovis aries PDE9A*. Meanwhile, it was closely associated with the mouse and rat *PDE9A*, with the *Homo sapiens* forming a separate group, while the non-mammalian species formed an even more distant group.

### 2.2. Expression Analysis of the Bovine PDE9A Gene

**Figure 2:**
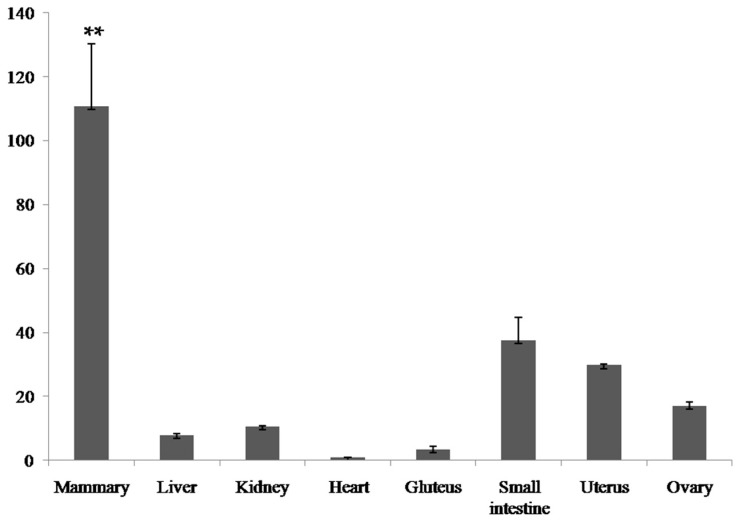
Relative quantification of the *PDE9A* gene in eight tissues. ** above the bars indicates significant difference between tissues (*p* < 0.01).

Tissue distribution analysis of bovine *PDE9A* was determined by quantitative real-time PCR and the relative expression results were normalized by internal *GAPDH* expression. As shown in Figure 2, *PDE9A* was widely expressed in all these detected tissues, with higher expression level in mammary gland, moderately expressed in small intestine, uterus and ovary, and only slightly expressed in liver, kidney, heart and gluteus. The highest mRNA expression level of *PDE9A* gene in mammary gland indicated that it was most likely related to the formation of milk related traits. As observed in previous investigations, some identified functional genes for milk production traits showed the same specially high expression patterns in lactating mammary tissue of mammals, that is, DGAT1 [14], ABCG2 [15] and SCD1 [16].

### 2.3. SNP Identification and Selection

Sequence analysis revealed that a total of 11 SNPs were found in the *PDE9A* gene using the pooled DNA of the eight unrelated bulls. Of them, six SNPs were in the 5′ regulatory region, two in intron14 and three in 3′ regulatory region. Considering the positions and functions of these polymorphisms, six SNPs in the 5′ regulatory region were finally chosen and genotyped for further association analysis. These selected SNPs were all in Hardy-Weinberg equilibrium (chi-square test, *p* > 0.05), and their genotypic and allelic frequencies are shown in Table 2.

**Table 2:** Genotypes and allelic frequencies of the six SNPs in the 5′ regulatory region of *PDE9A.*

SNP ID	GenBank No.	Allele Substitution	Genotypes	Genotypic Frequencies	Alleles	Allelic Frequencies
c.-2012 T>C	rs42140305		TT	0.40	T	0.64
		T>C	CT	0.48		
			CC	0.12	C	0.36
c.-2005 A>G	rs381951806		AA	0.56	A	0.74
		A>G	AG	0.37		
			GG	0.07	G	0.26
c.-1762 T>C	rs136803034		TT	0.37	T	0.61
		T>C	CT	0.49		
			CC	0.14	C	0.39
c.-1621 T>G	rs133033048		TT	0.52	T	0.72
		T>G	GT	0.38		
			GG	0.10	G	0.28
c.-1376 G>A	rs210080144		GG	0.65	G	0.81
		G>A	AG	0.32		
			AA	0.03	A	0.19
c.-724 A>G	rs135748250		AA	0.38	A	0.64
		A>G	AG	0.52		
			GG	0.10	G	0.36

### 2.4. Association Analyses

#### 2.4.1. Single Locus-Based Regression Analyses

Associations between the six SNPs and estimated breeding values (EBVs) of five milk production traits are shown in Table 3. Here, all these SNPs were statistically significant with protein yield (PY) (*p* < 0.0001~0.0077) and milk yield (MY) (*p* < 0.0001~0.0015; except for c.-1621 T>G). Meanwhile, the c.-1762 T>C, c.-1376 G>A and c.-724 A>G were strongly associated with fat yield (FY) (*p* = 0.0016~0.0009). After Bonferroni correction for multiple testing, these identified associations remained significant. Therefore, our results provide a valuable primary evidence for identifying candidate genes in dairy cattle.

Similarly, additive and allele substitution effects were observed for the three associated traits at all six loci as well (*p* < 0.05 ~ *p* < 0.01). The results of additive effects were mostly in consistent with those of allele substitution effects, which provided strong evidence for accuracy of additive genetic variance based on phenotypic individual differences. As for the dominant effect, only c.-1621 T>G reached significance on PY (*p* < 0.01), indicating that the genetic value for GT is not exactly the average of the genetic value of GG and TT. The estimated effects of the six SNPs on five traits are shown in Table 4.

**Table 3:** Associations of SNPs with EBVs for five milk production traits (least squares mean ± standard error).

SNP	Genotype	MY	FY	FP	PY	PP
c.-2012 T>C	CC (59)	531.94 ± 102.58 ^A^	11.44 ± 4.38	−0.05 ± 0.046	15.89 ± 3.06 ^A^	−0.006 ± 0.015
	CT (239)	363.53 ± 75.15 ^A^	3.62 ± 3.39	−0.07 ± 0.035	10.82 ± 2.37 ^A,B^	−0.002 ± 0.010
	TT (198)	192.65 ± 78.00 ^B^	0.91 ± 3.49	−0.04 ± 0.036	7.10 ± 2.44 ^B^	0.012 ± 0.011
	*p* Value	0.0002 **	0.0115	0.5315	0.0009 **	0.2303
c.-2005 A>G	AA (275)	242.80 ± 74.98 ^A^	6.24 ± 3.36	0.01 ± 0.023	9.72 ± 2.35 ^a^	0.003 ± 0.005
	AG (183)	361.60 ± 79.38 ^A,B^	6.77 ± 3.50	0.01 ± 0.030	12.42 ± 2.45 ^a,b^	0.003 ± 0.006
	GG (38)	596.23 ± 118.54 ^B^	11.44 ± 5.11	0.01 ± 0.034	18.84 ± 3.58 ^b^	−0.014 ± 0.012
	*p* Value	0.0015 **	0.4639	0.7746	0.0048 *	0.3535
c.-1762 T>C	CC (71)	620.74 ± 97.86 ^A^	15.84 ± 4.20 ^A,a^	−0.05 ± 0.044	19.10 ± 2.94 ^A^	−0.009 ± 0.015
	CT (240)	432.42 ± 75.59 ^A^	7.81 ± 3.41 ^b^	−0.06 ± 0.035	14.93 ± 2.39 ^A^	0.006 ± 0.010
	TT (185)	237.05 ± 77.76 ^B^	3.67 ± 3.47 ^B,b^	−0.04 ± 0.036	9.35 ± 2.43 ^B^	0.012 ± 0.011
	*p* Value	<0.0001 **	0.0013 **	0.6539	<0.0001 **	0.2924
c.-1621 T>G	GG (48)	500.63 ± 110.74	11.59 ± 4.68	−0.04 ± 0.050	17.44 ± 1.19 ^A,a^	0.013 ± 0.017
	GT (186)	240.61 ± 77.84	4.37 ± 3.48	−0.03 ± 0.036	8.99 ± 1.69 ^B,b^	0.013 ± 0.011
	TT (262)	312.57 ± 74.72	6.77 ± 3.38	−0.03 ± 0.034	10.39 ± 0.92 ^b^	0.005 ± 0.010
	*p* Value	0.0279	0.1578	0.9389	0.0077 *	0.6157
c.-1376 G>A	AA (14)	113.67 ± 167.77 ^A^	−2.48 ± 6.82 ^A^	−0.05 ± 0.075	3.64 ± 4.77 ^A^	0.007 ± 0.026
	AG (162)	158.20 ± 79.24 ^A^	1.22 ± 3.52 ^A^	−0.03 ± 0.036	4.86 ± 2.47 ^A^	0.003 ± 0.011
	GG (320)	459.43 ± 73.27 ^B^	9.06 ± 3.33 ^B^	−0.05 ± 0.033	14.71 ± 2.33 ^B^	0.004 ± 0.010
	*p* Value	<0.0001 **	0.0009 **	0.6907	<0.0001 **	0.9834
c.-724 A>G	AA (185)	547.23 ± 79.37 ^A^	10.08 ± 2.93 ^A^	−0.07 ± 0.036	16.55 ± 2.49 ^A^	−0.003 ± 0.011
	AG (262)	282.30 ± 73.85 ^B^	5.26 ± 2.75 ^AB^	−0.03 ± 0.034	9.65 ± 2.34 ^B^	0.009 ± 0.010
	GG (49)	36.21 ± 106.47 ^B^	−3.01 ± 3.37 ^B^	−0.03 ± 0.048	0.50 ± 3.15 ^C^	0.001 ± 0.016
	*p* Value	<0.0001 **	0.0016 **	0.2956	<0.0001 **	0.3851

Bonferroni corrected significant level at *p* < 0.05 and *p* < 0.01 were 0.0083 and 0.0017, respectively; * *p* indicates the significant association after Bonferroni correction for multiple testing at the significance level of 0.05. ** *p* indicates the significant association after Bonferroni correction for multiple testing at the significance level of 0.01.; **^a,b^** within the same column with different superscripts means *p <* 0.05, **^A,B,C^** means *p <* 0.01.

**Table 4:** Genetic effects of these SNPs on the five traits in dairy cattle. Notes: * *p* < 0.05; ** *p* < 0.01.

Locus	Gene Effects	MY	FY	FP	PY	PP
c.-2012 T>C	Additive (a)	169.64 **	5.26 **	−0.006	4.39 **	−0.009
	Dominant (d)	1.24	−2.55	−0.022	−0.68	−0.005
	Allele substitution (α)	47.04 **	4.57 **	−0.012	4.21 **	−0.010
c.-2005 A>G	Additive (a)	−176.72 **	−2.60	0.013	−4.56 **	−0.005
	Dominant (d)	−57.91	−2.07	0.001	−1.86	0.001
	Allele substitution (α)	−204.52 **	−3.59	0.014	−5.45 *	−0.005
c.-1762 T>C	Additive (a)	191.84 **	6.09 **	−0.005	4.87 **	−0.011
	Dominant (d)	3.53	−1.94	−0.018	0.71	0.004
	Allele substitution (α)	192.65 **	5.64 **	0.009	5.03 **	−0.010
c.-1621 T>G	Additive (a)	94.03	2.41	−0.007	3.53 **	0.004
	Dominant (d)	−165.99 *	−4.81 *	0.009	−4.93 **	0.004
	Allele substitution (α)	24.31	0.39	−0.003	1.46 **	−0.006
c.-1376 G>A	Additive (a)	−172.88 *	−5.77 *	0.001	−5.54 **	0.001
	Dominant (d)	−128.34	−2.08	0.021	−4.32	−0.003
	Allele substitution (α)	−252.53 **	−7.06 **	0.014	−8.22 **	−0.001
c.-724 A>G	Additive(a)	255.51 **	1.90 **	−0.021	8.03 **	−0.002
	Dominant (d)	−9.42	2.37	0.017	1.12	0.010
	Allele substitution (α)	252.87 **	2.28 **	−0.016	8.34 **	0.001

(a), (d), (α), means Additive, Dominant, Allele substitution effects, respectively; * the bold number means significant at *p* < 0.05; ** the bold number means significant at *p* < 0.01.

#### 2.4.2. Haplotype Regression Analyses

**Figure 3:**
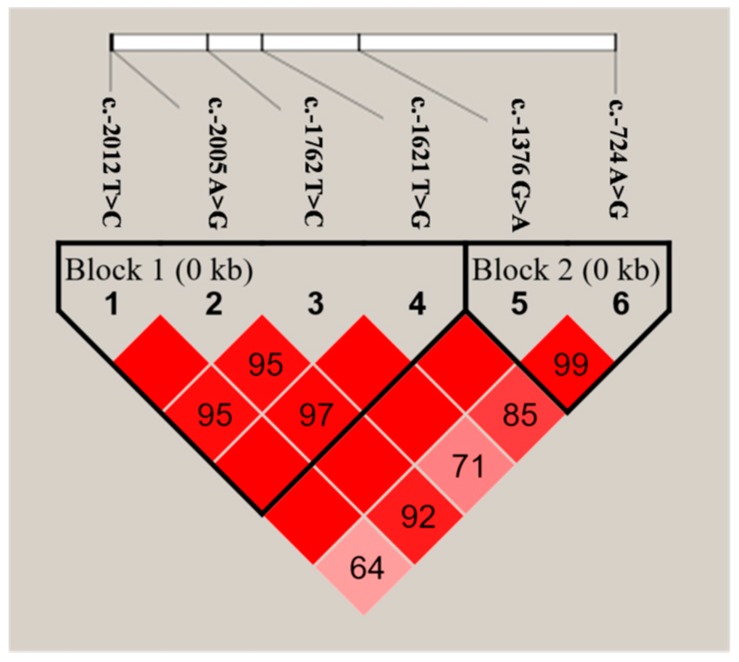
Linkage disequilibrium (LD) among the six SNPs in *PDE9A*. The value for *r*^2^ between each selected SNP is presented in each box.

Pair-wise D’ values and inferred haplotype blocks were shown in Figure 3. For the six SNPs, two adjacent blocks were identified. Block 1 consisted of four SNPs, which formed four haplotypes in the studied population. The major haplotypes CACT, TATT and TGTG accounted for the frequency of 34.8%, 31.9% and 25.7%, respectively. However, the pooled haplotypes (which with frequency <5% were pooled into a single group) occurred at the frequency of 7.6%. Block 2 was composed of two SNPs, which formed three haplotypes.

The single-marker analysis is usually thought to be noisy and less powerful due to lack of the simultaneous use of multi-point information [17,18]. Haplotype analysis, unlike single-marker analysis, is more likely to have significant effects on traits [19,20]. As shown in Table 5, the haplotypes in the two blocks both showed a significant association with MY, FY and PY. The results of haplotype association analyses were mostly consistent with those of the single-locus, which provided strong evidence for the associations between these SNPs and haplotypes with milk production traits.

**Table 5:** Main haplotypes of the *PDE9A* gene, their frequencies and associations with EBVs of five milk production traits.

Haplotypes	c.-2012 T>C	c.-2005 A>G	c.-1762 T>C	c.-1621 T>G	c.-1376 G>A	c.-724 A>G	Frequency (%)	MY (*p*-Value)	FY (*p*-Value)	PY (*p*-Value)
CACT	C	A	C	T			34.8	<0.0001 **	0.0035 **	<0.0001 **
TATT	T	A	T	T			31.9			
TGTG	T	G	T	G			25.7			
Pooled Haplotypes *	T	A	C	T			7.6			
	T	A	T	G						
	C	A	T	T						
GA					G	A	63.8	<0.0001 **	0.0045 **	<0.0001 **
AG					A	G	19.0			
GG					G	G	17.2			

* single group; * *p* indicates the significant association after Bonferroni correction for multiple testing at the significance level a = 0.05; ** *p* indicates the significant association after Bonferroni correction for multiple testing at the significance level a = 0.01.

### 2.5. Functional Prediction of the Allele-Dependent TFBS

**Figure 4:**
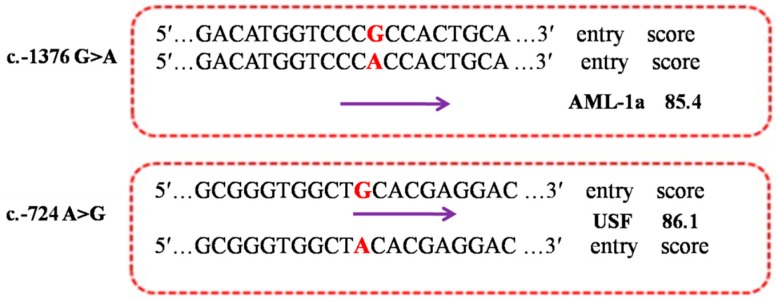
Prediction the alteration of TFBSs in the mutations c.-1376 G>A and c.-724 A>G by using TFSEARCH. The letters in red indicate the position of mutations; the arrows mean the direction of TFBSs.

As the most important mechanisms, the mutations in the regulatory region of a gene can affect transcription rate by changing the transcription factor binding sites (TFBSs) [21]. In view of the importance of these mutations, our study has highlighted the functions of these variants and their involvement in gene expression. Bioinformatic analyses using the TFSEARCH software revealed that the two regulatory SNPs, c.-1376 G>A and c.-724 A>G in *PDE9A* were predicted to change the binding site of the corresponding transcript factor acute myeloid leukemia 1 (AML-la) and upstream stimulatory factor (USF), respectively (Figure 4). USF family proteins are well known as ubiquitously expressed transcription factors with inhibition effect [22]. Functional analysis in cultured mammalian cells indicated that USFs are involved in the gene networks of cell proliferation [23]. However, none of reports on the biological function of AML-la have been found.

### 2.6. Expression Regulation of the Mutations in PDE9A Gene

**Figure 5:**
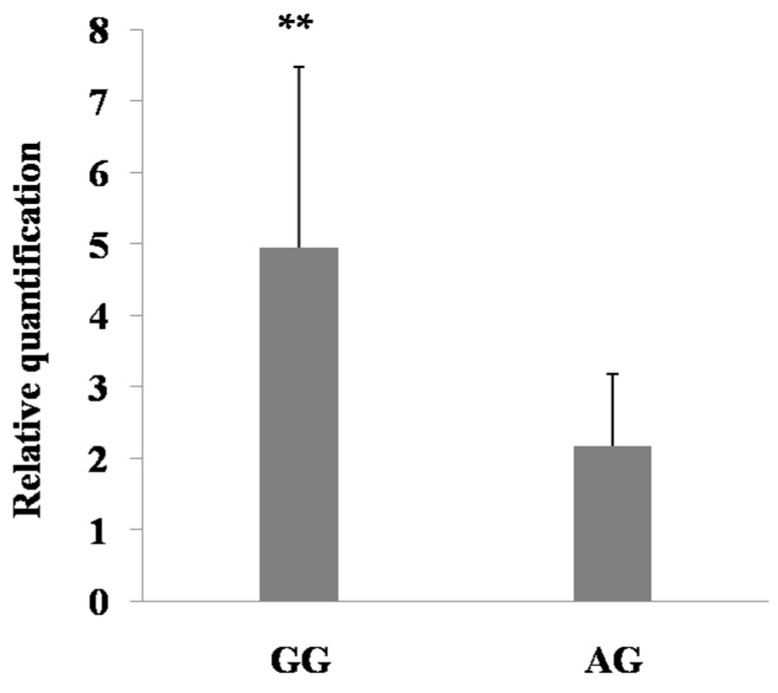
mRNA expression of c.-1376 G>A among the different genotypes in the mammary gland of lactating Holstein cows. Bars represent (mean value ± standard error) (*n* = 4). ** *p* < 0.01.

**Figure 6:**
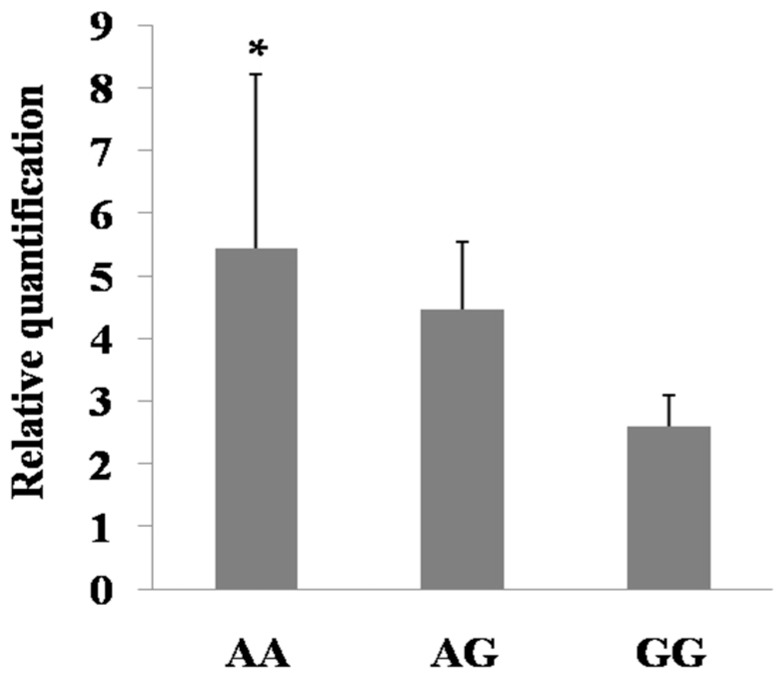
mRNA expression of c.-724 A>G among the different genotypes in the mammary gland of lactating Holstein cows.Bars represent mean ± SE (*n* = 5, 2, and 1, respectively). * *p* < 0.05.

To investigate potential regulation of the SNPs in 5′ regulatory region, the mRNA expression of *PDE9A* was measured in mammary glands based on the genotypes for both c.-1376 G>A and c.-724 A>G. The results showed that the mRNA levels with GG genotype of c.-1376 G>A and AA genotype of c.-724 A>G were higher than those of the other genotypes in mammary gland of Holstein cows in lactation (*p* < 0.01, *p* < 0.05; Figure 5 and Figure 6), respectively. In this study, the higher expression levels of *PDE9A* are also consistent with the higher EBVs for milk production traits. Hence, we hypothesized that the effects of such SNPs on the milk production traits may be partly induced by their regulatory role on the expression of *PDE9A*.

In this study, we demonstrated that significant associations exist between *PDE9A* variants and milk production traits in Chinese Holstein cows. To find more evidence for such associations, we compared our results with the known major gene and previously reported QTL and GWAS data. *PDE9A* is 2.8~4.0 Mb away from the three significant SNPs for fat yield, fat percentage and protein percentage with *p* value of 1.58 × 10^−6^~8.26 × 10^−7^ reported by the same GWAS [24]. It is also located within the QTL region of 127.4~148 cM for fat yield detected in a German Holstein cattle population [25].

As a novel candidate for milk production traits, *PDE9A* is merely studies in humans and mice until now [26,27]. *PDE9A* is a cGMP-specific enzyme and can activate cGMP-dependent signaling pathways by influencing intracellular cGMP levels [28]. As an important signaling molecule within cells, cGMP regulates cell growth, adhesion and energy homeostasis in normal physiological conditions [29]. Meanwhile, cGMP may be involved in the regulation of lipogenesis in mouse mammary gland [30]. Interestingly, a study showed that cGMP also participated in milk formation in goat mammary gland (unpublished data). In addition, *PDE9A* was reported to be a major regulator of basal cGMP levels in human breast cancer cells and inhibition of phosphodiesterases elevated intracellular cGMP [8] and involved in the negative feedback control of cyclic nucleotides pathways [31]. According to the phylogenetic tree, the bovine *PDE9A* was found to be phylogenetically closest to *Capra hircus* and *Ovis aries PDE9A*. Meanwhile, it was closely associated with the mouse and rat *PDE9A*.Considering the conservation of *PDE9A* gene in mammalian species, we inferred that the *PDE9A* gene might potentially influence the formation of milk traits or mammary physiological processing. In this study, the higher expression levels of *PDE9A* are also consistent with the higher EBVs for milk production traits.

In association analyses, the EBVs of daughters were used as phenotypic observations. EBVs are usually used as dependent variables in association analyses concerning milk production traits in dairy cattle [5,32]. We have also compared the results denoted by EBVs and phenotypic values for association analyses in our study, which demonstrated that the findings based on the two different variables were basically overlapped. Consequently, we herein used the EBVs for association analysis. As the haplotype analysis including multiple marker information was thought more powerful than single marker analysis [33], we further performed the haplotype association analysis to detect the association of PDE9A variants with milk production traits. It is obviously clear that the results of single-locus and haplotype association analysis were totally consistent. Accordingly, our findings could be used as genetic marker in genomic selection program of dairy cattle.

Compared to conventional breeding, the marker-assisted selection (MAS) can accelerate genetic gains dramatically [34]. Based on our findings herein, significant associations between polymorphisms in *PDE9A* and dairy production traits were revealed in dairy cattle. Specifically, we can incorporate the information of the *PDE9A* into selection program for practical use of these variations in breeding programs. Further biological analysis should be conducted in order to check the effects of such polymorphisms and validate its function on milk production traits before apply as markers for marker-assisted selection in the Chinese Holstein population. In addition, the promising variants of the *PDE9A* gene can be applied to increase the proportion of the marker, which is positively associated with the milk production traits.

## 3. Experimental Section

### 3.1. Bioinformatic Analysis of PDE9A Protein

*PDE9A* protein homology between the bovine and other species were obtained from Genbank using the BLAST program [35]. Multiple alignments were conducted using Clustal X Version 2.0. Moreover, a phylogenetic tree of PDE9A protein was further constructed using the Molecular Evolutionary Genetics Analysis (MEGA5.1) software [36]. Based on the neighbor-joining method, the number noted at branches indicates the percentage of times that a node was supported in 1000 bootstrap pseudo replications.

### 3.2. Gene Expression Assays of PDE9A Gene

To further confirm the potential function of *PDE9A*, we conducted gene expression analyses aiming at different tissues and different genotypes, respectively. Eight lactating Chinese Holstein cows in their 2nd/3rd lactation were selected from the Beijing F dairy farm center. Samples of eight tissues, including mammary gland, heart, uterus, kidney, liver, gluteus, ovary and small intestine, were collected for each cow and then stored at liquid nitrogen.

Total RNA was isolated from tissues using Trizol reagent (Invitrogen, Carlsbad, CA, USA) according to the manufacturer’s protocols. Subsequently, RNA extracts were incubated with RNase-free DNase I for 30 min at 37 °C to remove DNA contamination. Reverse transcriptase reaction was conducted using PrimerScriptH RT reagent Kit (TaKaRa Biotechnology Co., Ltd., Dalian, China). Real-time PCR using SYBR green fluorescence (Roche, Penzberg, Germany) was performed with a volume of 15 μL containing 7.5 μL SYBR Green Mixture, 2 μL template of cDNA, 0.375 μL of each primer, 4.75 μL distilled water. The PCR conditions: denaturation at 95 °C for 10 s; amplification 55 cycles at 95 °C for 10 s, 59 °C for 15 s, followed by 72 °C for 25 s. Quantitative real-time PCR primers of *PDE9A* and *GAPDH* were shown in Table S1. All measurements were performed in triplicate and the relative gene expression was normalized by the *GAPDH* with 2^−ΔΔ*C*t^ method, as described previously [16].

To further detect the effects of variants of *PDE9A*, the mRNA expression of mammary glands with different genotypes at functionally important mutations were also analyzed. On the basis of association analysis results and genotypes of the two SNPs (c.-1376 G>A, c.-724 A>G) in the 5′ regulatory region, the *PDE9A* mRNA expression of mammary glands in eight daughters among different genotypes were also detected. The results of mRNA expression were performed by GLM procedure of the SAS 9.1.3 software (SAS Institute, Cary, NC, USA).

### 3.3. Resource Population and DNA Extraction

The resource population was chosen from 14 dairy farms in the Sanyuanlvhe Dairy Farming Center (Beijing, China), where routine standard performance test (Dairy Herd Improvement system, DHI, Beijing, China) have been carried out since 1999. A daughter design was employed in this study. A total of 506 Chinese Holstein cows, which were daughters from 8 sire families, were selected to construct a single population in this study. Estimated breeding values (EBVs) of recorded cattle for each milk production trait (*i.e.*, milk yield, fat yield, protein yield, fat percentage, and protein percentage) were provided by the Dairy Association of China (DAC) using the genetic parameters estimated based on the complete DHI data of Chinese dairy cattle population.

Genomic DNA (gDNA) was isolated from whole blood samples of the daughters by Blood DNA Kit according to the manufacturer’s instructions (Tiangen Biotech Co., Beijing, China) and frozen semen of the 8 bulls using a standard phenol-chloroform method and stored at −20 °C. The quality and quantity of extracted genomic DNA were measured with NanoDrop™ Spectrophotometer (ND-2000c) (Thermo Scientific, Chelmsford, MA, USA).

### 3.4. SNP Identification and Genotyping

Genomic DNA samples of 8 Holstein bulls were selected to construct a pool DNA with identical DNA concentration (50 ng/mL).Using software Primer3.0, according to the genomic sequence of *PDE9A* (AC000158.1), a total of 31 pairs of primers were designed to amplify their entire coding regions, partial introns as well as regulatory regions of 5′ and 3′, respectively (Table S2). PCR amplification was performed in a volume of 50 μL, containing 2 μL of pooled DNA, 20 pmol of each forward and reverse primer, 25 μL of 2× MightyAmp buffer, and 1.25 U of MightyAmp DNA polymerase (Takara Biotechnology Co., Ltd., Dalian, China). The Cycling parameters were as follows: denaturation 95 °C for 10 min, followed by 36 cycles of 95 °C for 30 s, 60 °C for 30 s, 30 s at 72 °C, and with a final extension completed at 72 °C for 7 min. Then, PCR products of each fragment were sequenced by an ABI3730XL sequencer (Applied Biosystems, Foster City, CA, USA).

SNaPshot assay was applied for genotyping of the 6 SNPs in the 5′-regulatory region of *PDE9A* of each cow with Peak ABI PRISM^®^ SNaPshot™ Multiplex Kit (Applied Biosystems, Foster City, CA, USA). In follow-up analyses, scanner software v1.0 (Applied Biosystems) was used to identify the genotype of each individualforall the SNPs.

### 3.5. Haplotype Analysis

To further estimate the LD extent for each pair of variants genotyped in the *PDE9A* gene, the software Haploview 4.2 (Broad Institute of MIT and Harvard, Cambridge, MA, USA) was performed [14]. Accordingly, haplotype blocks were also explored with the subject genotype data via Haploview based on the criterion of D’. In this analysis, the haplotype with frequency >5% was considered as a single haplotype, and those with frequency <5% were pooled into a distinguishable group. Association between each haplotype and the five milk production traits was performed in subsequent analyses.

### 3.6. Association Analysis

Pedigree information of the resource population was traced back for three generations to create the numerator relationship matrix. Kinship matrix (A-matrix) was constructed using MATLAB version 7.11 (R2010b). Based on the estimated variance-covariance matrices, the association of SNPs and haplotypes of *PDE9A* with the 5 milk production traits were evaluated using the mixed procedure in SAS 9.1.3 (SAS Institute, Cary, NC, USA) with the following mixed animal model [31]. A linear mixed model was fitted as follows:

(1) y=1μ+bx+Za+e

where **y** is the vector of phenotypes (EBV) of all the cows, μ is the general mean. **x** is the fixed effect vector of the SNP genotype or haplotype combination, *b* is the regression coefficient, Z is the incidence matrix of **a**, **a** is the vector of residual additive effects with distribution of **a~*N*** (0, $A\delta_{a}^{2}$) (**A** is the polygenic relationship matrix among all the individuals, $\delta_{a}^{2}$ is the additive genetic variance), and finally **e** denotes the residual errors with distribution of **e~*N*** (0, $W\delta_{e}^{2}$) (**W** is a diagonal matrix with the diagonal elements equal to 1/REL_ij_ and $\delta_{\text{e}}^{2}$ is the residual error variance).

The additive (a), dominance (d) and allele substitution (α) effects were estimated using the equation: a = (AA − BB)/2, d = AB − (AA + BB)/2 and α = a + d (q − p), where AA or BB indicates the two homozygous genotypes, AB indicates heterozygous genotype, p and q are the allele frequencies of corresponding locus. In addition, bonferroni correction was performed for multiple *t*-testing in subsequent analyses.

### 3.7. Bioinformatics Analysis of Transcription Factor Binding Sites (TFBSs)

The online software TFSEARCH [37] was used to predict the putative differential TFBSs according to the mutations with standard settings for the highest similarity of the 6 regulatory SNPs in *PDE9A* gene.

## 4. Conclusions

In summary, we validated the significant associations of *PDE9A* geneidentified in previous GWA study with milk production traits and showed that mRNA expression levels of the *PDE9A* gene were highest in mammary gland tissue of Chinese Holstein. Additionally, two SNPs in 5′ regulatory region were identified to be involved in the regulation of gene expression. These findings strongly suggest that the associated variants could be used as molecular markers in advanced marker-assisted selection to facilitate the breeding of desired production traits in the Chinese Holstein population.

## Supplementary Materials

Supplementary materials can be found at http://www.mdpi.com/1422-0067/16/11/25976/s1.
