# Supplementary material for: Validation of PDE9A Gene Identified in GWAS Showing Strong Association with Milk Production Traits in Chinese Holstein
Source: Int J Mol Sci. 2015 Nov 5;16(11):26530–42. doi: 10.3390/ijms161125976 (PMC4661835; doi:10.3390/ijms161125976)
Supplement: Supplementary file 1 [file ijms-16-25976-s001.pdf]

International Journal of
Molecular Sciences

MDPI

Supplementary Information

Table S1. Primers used for the amplification of bovine *PDE9A* and *GAPDH* genes by qPCR.

Gene	Forward Primer	Reverse Primer	Tm	Product Length
PDE9A	CCCGATGTTTGAGACAGTGA	CTCTGCTTTCTTCAGGCACA	60	215 bp
GAPDH	AGATGGTGAAGGTCGGAGTG	CGTTCTCTGCCTTGACTGTG	60	189 bp

Table S2. Primers used for SNP identification in the *PDE9A* gene.

Location	Forward Primer Sequence	Reverse Primer Sequence	Product Length
5′ region	TCTGCTCAGCCTCCTGTATG	CTGGGCATTCAGTGGAGAAT	434 bp
5′ region	AGCAAGGGCAGCACTGATAG	AAGATTGGAGCGAGTGCTGT	461 bp
5′ region	AACAGCACTCGCTCCAATCT	GGATGGTCCAGCATATGTCA	428 bp
5′ region	CATTCCTTGAGAGCCAGTCG	TTCAGTGCCTGTTGCTTTTG	417 bp
5′ region	GCAAACCCCAGACATTCAAC	TGTGACCCAAGCACCACTTA	533 bp
5′ region	TGGAAAAGGCGATCACTC	TCTCCTCCCCTCCCACTTTA	528 bp
exon1	CTGGCGTCGAGAAAGTACAG	CGTCGGAACTCAGCCTAATC	341 bp
exon2	TCTGCTCAGCCTCCTGTATG	CTGGGCATTCAGTGGAGAAT	504 bp
exon3	GTGCACCTGCAACCCTTAAT	TTGGTTTAAACGCGGATTTC	383 bp
exon4	GCAGAGGGGGTCCATTATTT	GGGCTTCTTGCGTTACAGTC	538 bp
exon5	ACCAGTCCTGCCTGCTTTTA	GGGAAGTGATGGATGAGAGC	442 bp
exon6	CGCTCTGTGTTCTGGTCTTG	CGTTGCGAGTTACTGGGTTA	527 bp
exon7	GATGAACAGGCGTGTGTCTG	CGCTTCCTCTCTTCTTCTGC	548 bp
exon8	CCATCAACTCTGGGGTTTGT	ACGATCTCCTGGCAGTAAGC	453 bp
exon9	GGTCAGTCCTGAAGCACTCC	TTTCTGAGCAGCCAGATCCT	482 bp
exon10	GTGCTGAAACCCCCTAGTCA	AGAGTGTCTGGTTGGGGATG	425 bp
exon11	AACCCCAGGAAGCAGGAA	CCACCTCCCCAACAGCAG	308 bp
exon12	CTTCATCCCCCTGTGTCATT	GTCATGGGTGTGTGAACGTC	487 bp
exon13	AGTTCCTGGCACAGCAGAGT	CCAAGCACACACCTCTTCCT	500 bp
exon14	TGGCATGAGGTCTGAATGAA	CTGGATTCTGCCCTCTGTTC	550 bp
exon15	GAGGGGTCACAGCAAAGTTC	CCTGCCTTGTGTGTCTCAAA	418 bp
exon16	ATCTGTGATGCGGGTGATTC	CATGAGGCTATGTGGGTGTG	571 bp
exon17	GTGACCCTGGACAGAAGCAT	GTGGCAGAGGCAGTGATGT	428 bp
exon18	GAGCTGACCCACAAAAGAGG	CGGGAGAGAGTAACGGACAG	483 bp
exon19	CCTCAGAGCCCCAATCTCTA	GGCACTCAGCAAGTCACTCA	507 bp
3′ region	AACACTGTGCCTTCCTCACC	CAGTCCACCCAGAACATCCT	432 bp
3′ region	AGGATGTTCTGGGTGGACTG	CAGAGTTTGGTGAGGCAGGT	576 bp
3′ region	CTCCTGTGCTGGCGTTTTAT	TAAGCACGCGTATGTTGCAT	469 bp
3′ region	CTGAGGCCCCATACACACAT	CCTGTTCCAGGACTTGGTGT	479 bp
3′ region	CTGCCTGACTGGGTGTAGGT	CTGGCAGCTTCTCTGCAACT	466 bp
3′ region	CCCCTTCCTGAGTCTCCTCT	GGAGGATTTGTTGGGGATTT	474 bp
